# Neuronal F-Box protein FBXO41 regulates synaptic transmission and hippocampal network maturation

**DOI:** 10.1016/j.isci.2022.104069

**Published:** 2022-03-18

**Authors:** Ana R.A.A. Quadros, Rocío Díez Arazola, Andrea Romaguera Álvarez, Johny Pires, Rhiannon M. Meredith, Ingrid Saarloos, Matthijs Verhage, Ruud F. Toonen

**Affiliations:** 1 Department of Functional Genomics, Vrije Universiteit (VU) Amsterdam, de Boelelaan 1085, 1081 HV Amsterdam, The Netherlands; 2 Department of Integrative Neurophysiology, Vrije Universiteit (VU) Amsterdam, de Boelelaan 1085, 1081 HV Amsterdam, The Netherlands; 3 Department of Clinical Genetics, Center for Neurogenomics and Cognitive Research (CNCR), Vrije Universiteit (VU) Amsterdam, de Boelelaan 1085, 1081 HV Amsterdam, The Netherlands

**Keywords:** Molecular neuroscience, Developmental neuroscience, Cellular neuroscience

## Abstract

FBXO41 is a neuron-specific E3 ligase subunit implicated in epileptic encephalopathies. *Fbxo41* null mutant (KO) mice show behavioral deficits and early lethality. Here, we report that loss of FBXO41 causes defects in synaptic transmission and brain development. Cultured *Fbxo41* KO neurons had normal morphology and showed no signs of degeneration. Single-cell electrophysiology showed a lower synaptic vesicle release probability in excitatory neurons. Inhibitory neurons exhibited reduced synaptophysin expression, a smaller readily releasable pool, and decreased charge transfer during repetitive stimulation. In *Fbxo41* KO hippocampal slices at postnatal day 6, the dentate gyrus was smaller with fewer radial-glial-like cells and immature neurons. In addition, neuronal migration was delayed. Two-photon calcium imaging showed a delayed increase in network activity and synchronicity. Together, our findings point toward a role for FBXO41 in synaptic transmission and postnatal brain development, before behavioral deficits are detected in *Fbxo41* KO mice.

## Highlights

• FBXO41 controls synaptic transmission in excitatory and inhibitory neurons differently
• *Fbxo41* KO hippocampi are smaller, with fewer radial-glial-like cells and immature neurons
• *Fbxo41* KO hippocampus shows neuronal migration and network maturation defects
• FBXO41 plays a critical role in neuronal transmission and postnatal brain development

## Graphical abstract

**Figure:**
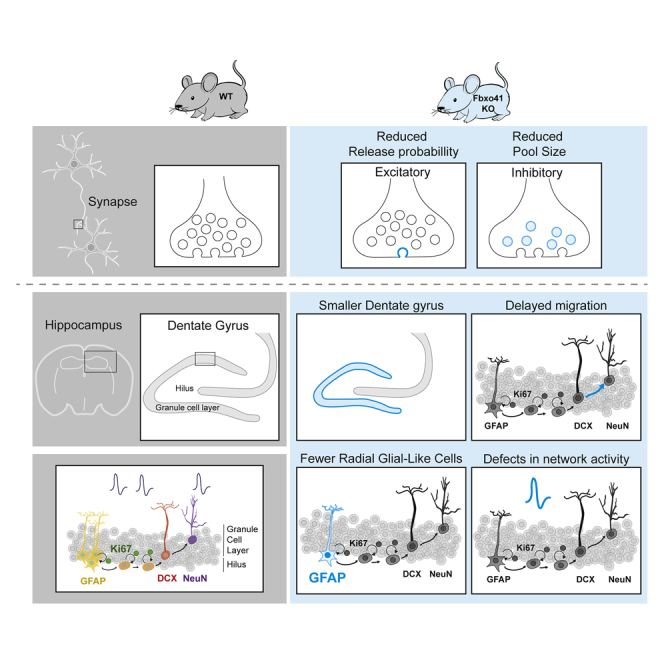

## Introduction

The dentate gyrus (DG), the primary afferent pathway into the hippocampus, is important for memory formation, and its development has been extensively described (Altman and Bayer, 1990a, 1990b; Urban and Guillemot, 2014; Li and Pleasure, 2005). During embryonic to early postnatal stages, neural progenitors proliferate and migrate along the dentate migratory stream from the neuroepithelium to the dentate primordium, giving rise to three distinct layers: 1) the molecular layer occupied by dendrites of the dentate granule cells, 2) the granule cell layer (GCL) composed of densely packed granule cells, and 3) the hilus, containing several cell types and enclosed by the GCL (Altman and Bayer, 1990a, 1990b; Amaral et al., 2007; Li and Pleasure, 2005; Urban and Guillemot, 2014). After proliferation and migration, neurons integrate in networks with temporally and spatially fine-tuned activity patterns. These immature patterns of spontaneous activity during early development are believed to be fundamental for proper network maturation (reviewed in Kirkby et al., 2013). Perturbing key processes of DG development results in long-lasting deficits (reviewed in Ben-Ari, 2001; Kirkby et al., 2013). However, knowledge of the mechanisms that regulate network refinement in the DG is still incomplete.

FBXO41 is a brain-enriched, neuron-specific F-box protein that is the target-recognizing subunit of a multi-subunit SKP1/Cullin 1/F-box (SCF) E3 ligase complex (King et al., 2019; Mukherjee et al., 2015). *Fbxo41* mutations have been linked to neurological abnormalities both in humans and in mice: *Fbxo41* KO mice show neuronal migration defects in the cerebellum, signs of neurodegeneration, and severe motor deficits, resembling ataxia. Only 20% of *Fbxo41* KO mice survive beyond postnatal day 14 (Mukherjee et al., 2015). In addition, accumulation of FBXO41 at centrosomes disassembles primary cilia and impairs ciliary sonic hedgehog signaling (King et al., 2019), which is crucial for neuronal migration and brain development (reviewed in Guemez-Gamboa et al., 2014). In humans, a *de novo* heterozygous nonsense mutation in *FBXO41* was detected in exome-sequencing data of 356 patients with epileptic encephalopathies (Epi et al., 2013; Euro et al., 2014). Hence, Fbxo41 is critical for normal brain function. However, insight into underlying mechanisms is still limited.

Disturbed synaptic excitation-inhibition balance may underlie epileptic encephalopathies. To test this, we assessed synaptic transmission in cultured excitatory and inhibitory *Fbxo41* KO neurons. *Fbxo41* KO neurons had normal neurite length and synaptic density. However, neurotransmission was less efficient in excitatory and inhibitory neurons with a more prominent effect on inhibition. Fbxo41 depletion also affected hippocampal development: *Fbxo41* KO mice had a smaller DG with fewer cells that were already at P6. The number of GFAP-positive RGL cells and immature doublecortin-positive (DCX+) neurons were reduced in *Fbxo41* KO, whereas mature (NEUN+) neurons and dividing (Ki67+) cells were mostly unaffected. In addition, at P6, NEUN+ cells accumulated close to the hilus, indicating a migration defect. Finally, two-photon calcium imaging showed that development of network activity was delayed with less active and synchronous neuronal firing. Collectively, our data uncovers new roles for FBXO41: in its absence, neurotransmission is less efficient, especially in inhibitory neurons. In addition, DG size, composition, and network activity are affected. These defects may underlie the behavioral phenotype of *Fbxo41* KO mice.

## Results

### FBXO41 is expressed in most brain areas and its expression increases over time

**Figure 1:**
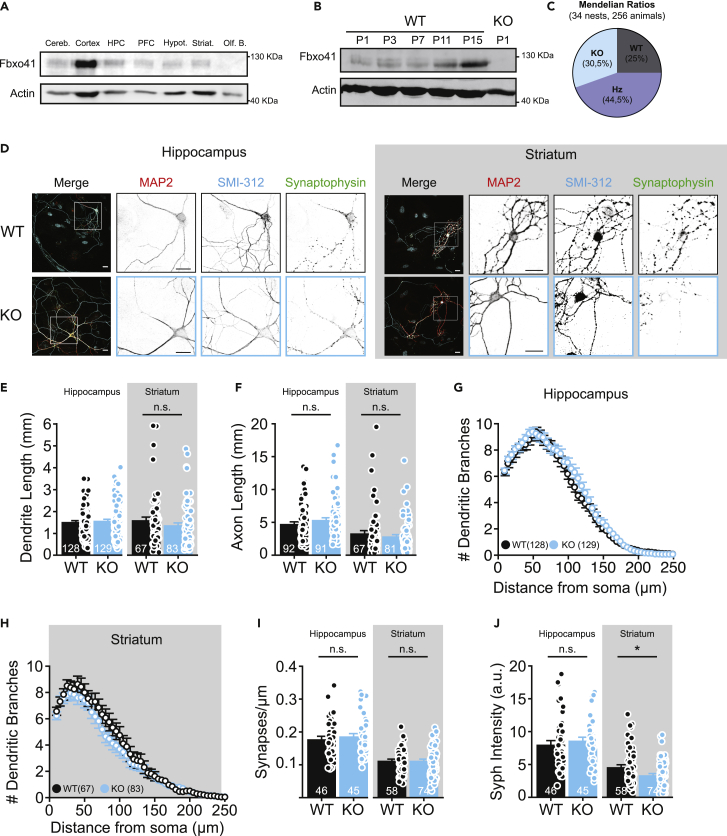
Fbxo41 KO hippocampal and striatal neurons have normal morphology with reduced synaptophysin expression in striatal neurons (A) Western blot from P1 WT mice showing FBXO41 expression in several brain areas, including the cerebellum (Cereb), cortex, hippocampus (HPC), prefrontal cortex (PFC), hypothalamus (Hypot), striatum (Striat), and olfactory bulb (Olf. B.). Actin was used as loading control. (B) Western blot showing increasing levels of FBXO41 in the hippocampus of WT mice from P1 to 15. Fbxo41 was not detected in the P1 *Fbxo41* KO hippocampus. (C) Mendelian ratios calculated by dividing the total number of animals from a certain genotype by the total number of animals. For calculation of these ratios, 256 animals were considered from a total of 34 nests. (D) Representative images and zooms of DIV14 hippocampal (left) and striatal (right, gray box) neurons stained for dendritic (MAP2), axonal (Smi-312), and synaptic (synaptophysin) markers from control (WT) and Fbxo41 null (KO) mice. Scale bar = 20 μm. (E and F) Dendrites and axons have normal length in the absence of Fbxo41, both in hippocampal (left) and striatal (right, gray box) neurons. (G and H) Dendritic arborization (Sholl analysis) is not altered in the absence of Fbxo41, neither in hippocampal (G) nor in striatal (H) neurons. (I) Synapse density is unaltered in hippocampal (left) or striatal (right, gray box) KO neurons. (J) The synaptic intensity of synaptophysin is similar between WT and KO hippocampal neurons (left), but is reduced in Fbxo41 KO striatal neurons (right, gray box). Data from 3–5 independent experimental weeks and represent average ±SEM. Numbers in bars are neurons, dots represent individual neuron data. For statistical details see Table S1.

To study the role of FBXO41 in neuronal morphology and synaptic transmission, we first assessed FBXO41 expression in the brain. FBXO41 was enriched in the cortex around birth (P1) and detected in several other brain regions, including the hippocampus and striatum, except for the olfactory bulb (Figure 1A). In addition, FBXO41 expression increased over time in the hippocampus (Figure 1B), similarly to what was previously reported in the cerebellum (Mukherjee et al., 2015). *Fbxo41* KO animals were born at the expected Mendelian ratio (Figure 1C). Therefore, FBXO41 is a developmentally expressed brain protein, which does not affect birth rates.

### FBXO41 deficiency does not affect neuronal morphology but reduces synaptophysin expression in striatal neurons

To test if FBXO41 is important for neuronal development, we assessed morphology of hippocampal excitatory and striatal inhibitory day *in vitro* (DIV) 14 neurons grown in isolation on glia micro-islands. Neurons were stained with dendritic (MAP2), axonal (SMI-312), and synaptic (synaptophysin) markers (Figure 1D). Neurite length (Figures 1E and 1F), dendritic complexity (Figure 1G and 1H), and synapse density (Figure 1I) were all similar between WT and *Fbxo41* KO in both neuronal populations. The intensity of synaptophysin puncta was normal in hippocampal but decreased in striatal neurons (Figure 1J).

### Neurotransmission is less efficient in the absence of FBXO41

**Figure 2:**
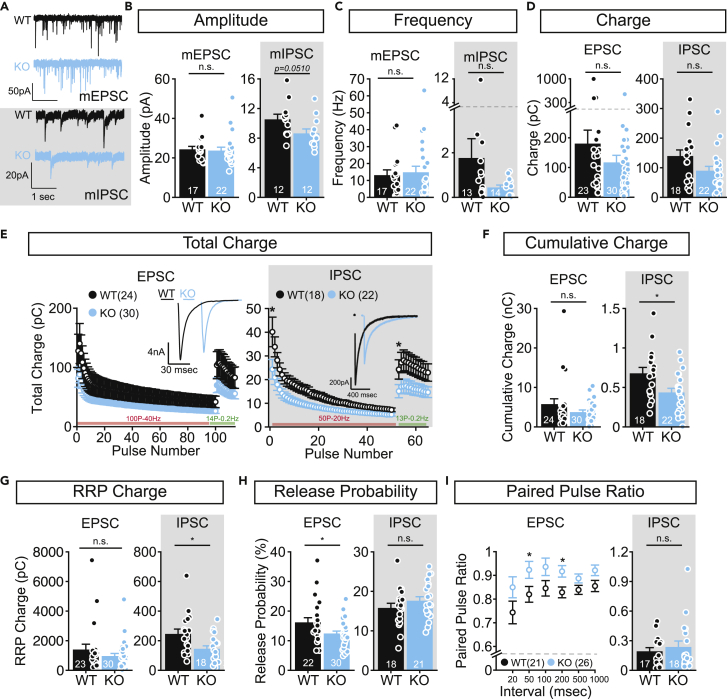
FBXO41 deficiency decreases vesicular release probability in glutamatergic hippocampal neurons and charge transfer during repetitive stimulation in GABAergic neurons (A) Typical traces of voltage-clamp recordings from isolated hippocampal excitatory (top, mEPSC) and striatal inhibitory (bottom, gray box, mIPSC) WT and *Fbxo41* KO neurons. (B and C) Spontaneous miniature amplitude (B) and frequency (C) are not significantly different in *Fbxo41* KO excitatory (left, mEPSC), or inhibitory (left, gray box, mIPSC) neurons compared to WT. (D) Average EPSC (left) or IPSC (right, gray box) charge is not significantly different in *Fbxo41* KO compared to WT neurons. (E) left: Total charge transfer during 100 action potentials at 40 Hz, followed by 14 action potentials at 0.2 Hz are similar in KO and WT glutamatergic neurons.Right: Total charge transfer during 50 action potentials at 20 Hz (which depletes the RRP in GABAergic neurons), followed by 13 action potentials at 0.2 Hz in *Fbxo41* KO and WT GABAergic neurons. The total charge of the first IPSC in this train and the first recovery pulse are significantly smaller in the KO compared to WT (see also Figure S1).Inset shows example traces of responses induced by single action potentials. (F) The total charge released during the trains depicted in E is not affected in excitatory neurons (left, EPSC), but is significantly reduced in inhibitory KO neurons (right, gray box, IPSC) compared to WT. (G) The RRP, assessed by back extrapolating the cumulative EPSC or IPSC charge in the trains depicted in E to the Y axis (see also Figures S1D and S1H), is smaller in inhibitory KO cells (right, gray box, IPSC), but unaffected in excitatory cells (left, EPSC). (H) The vesicular release probability calculated by dividing the total charge of the first single action potential by the total charge of the RRP is significantly lower in excitatory neurons lacking FBXO41 (left, EPSC), but unaffected in inhibitory neurons (right, IPSC). (I) Left: Paired pulse ratio assessed by stimulating neurons with two consecutive pulses with varying intervals is increased in excitatory *Fbxo41* KO neurons for pulses with intervals of 50, 200, and 1000 msec. Right: The paired pulse ratio assessed by stimulating neurons with two consecutive pulses with a 20 msec interval is not affected in inhibitory KO neurons. Data from 4 independent experimental weeks represent average ± SEM. Numbers in bars are neurons, dots represent individual neuron data. For statistical details see Table S1.

Next, we tested synaptic transmission in single-isolated glutamatergic hippocampal and striatal inhibitory neurons from Fbxo41 KO and wild type littermates using whole-cell voltage clamp electrophysiology (Bekkers and Stevens, 1991; Wierda et al., 2007). Spontaneous excitatory (mEPSC) or inhibitory (mIPSC) postsynaptic current amplitude and frequency were not significantly different between *Fbxo41* KO and WT (Figures 2A–2C). Evoked-EPSC and IPSC charge and amplitude were reduced in *Fbxo41* KO, but did not reach statistical significance (Figures 2D, S1A, and S1E). To test transmission during prolonged activity, glutamatergic neurons were stimulated with 100 action potentials at 40 Hz, a stimulus known to deplete the readily releasable pool (RRP; Rhee et al., 2002), followed by 14 recovery pulses at 0.2 Hz (Figures 2E and S1B). No significant differences were observed between WT and KO glutamatergic neurons in rundown kinetics, cumulative charge released during the train or RRP recovery (Figures 2E–2G and S1B–S1D). The size of the RRP, estimated by back-extrapolating the cumulative charge of the 40 Hz train (Figure S1D), was also similar between WT and KO neurons (Figure 2G). In striatal neurons, the RRP depletes upon 50 action potentials at 20 Hz (Figure S1F and Nair et al., 2013). Charge transfer during this stimulation was reduced in *Fbxo41* KO neurons (Figures 2E and 2F) without affecting rundown or recovery kinetics (Figure S1F). RRP size, estimated by back extrapolating the cumulative charge released during the 20 Hz train (Figure S1H), was smaller in striatal *Fbxo41* KO neurons (Figure 2G). Synaptic vesicle release probability, calculated by dividing the EPSC/IPSC charge by the total charge of the RRP, was reduced in excitatory *Fbxo41* KO neurons but remained unaffected in inhibitory ones (Figure 2H). Similarly, paired-pulse ratios, determined by two consecutive pulses at different time intervals, were higher in glutamatergic KO neurons at 50 and 200 millisecond (msec) intervals (Figure 2I) and were not affected in inhibitory neurons at 20 msec interval (Figure 2I). Hence, glutamatergic hippocampal neurons lacking FBXO41 have a decreased vesicular release probability while GABAergic KO neurons transfer less charge, both upon single and repetitive stimulation from a smaller RRP.

### Fbxo41 mice have a smaller dentate gyrus with fewer cells

**Figure 3:**
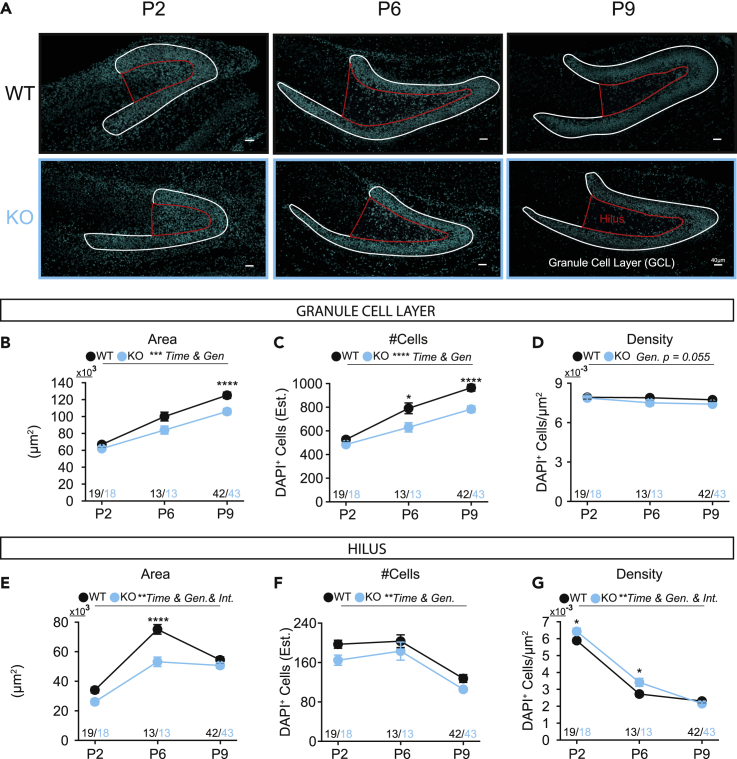
Fbxo41 KO animals have a smaller dentate gyrus Immunohistochemistry analysis of WT and Fbxo41 KO hippocampus at P2, 6, and 9. At P2 the GCL was defined as the cluster of cells accumulated at the DG, and that will later form the GCL (Nicola et al., 2015). (A) Example figures of DAPI staining in the DG of P2, P6, and P9 WT (top) and KO animals (bottom) with overlay showing the granule cell layer (GCL, white) and the hilus (red); scale bar 40 μm. Images without masks are depicted in Figure S2. (B) The granule cell layer (GCL) area, determined using DAPI staining, is smaller in Fbxo41 KO and increases with postnatal day in WT and KO. (C) The estimated number of DAPI positive cells in the GCL, calculated by dividing the total DAPI intensity in the GCL mask by the total intensity of a single DAPI^+^ cell, is lower in the hippocampus lacking FBXO41, and increases with postnatal day. (D) The DAPI^+^ cell density, number of cells per area, is not different between WT and KO, and is not affected by time. (E) The hilus area increases from postnatal day 2 to 10, and is significantly smaller at P6 in KO animals compared to the WT controls. (F) The estimated number of DAPI positive cells in the hilus decreases from P2 to P9 and is smaller in *Fbxo41* KO. (G) The DAPI^+^ cell density in the hilus decreases from P2 to P10, and is higher in KO compared to WT at P2 and P6. Data from 3 (P2 and P10) or 2 (P6) independent weeks. Data is represented as mean ± SEM. Numbers in figures are slices. For statistical details see Table S1.

To evaluate the role of FBXO41 in hippocampal development, we first assessed hippocampal architecture in brain slices of *Fbxo41* KO mice and WT littermates at three time points during early postnatal development (P2, P6, and P9). Gross hippocampal morphology, with clearly distinguishable CA1-3 and DG regions, was not affected in *Fbxo41* KO mice (Figure 3A), in line with previous observations (Mukherjee et al., 2015). To study DG architecture in more detail, we used the nuclear marker DAPI to determine the area and cell numbers of the GCL and hilus, which is surrounded by the GCL (Figures 3A and S2). In WT hippocampi, GCL area (Figure 3B) and number of DAPI^+^ nuclei (Figure 3C) — calculated by dividing total DAPI intensity by single cell intensity — increased from P2 to P9, whereas cell density — number of cells divided by total area — remained constant (Figure 3D). *Fbxo41* KO hippocampi showed a similar increase in GCL area and number of DAPI^+^ nuclei over time, but GCL area (Figure 3B) and the number of DAPI^+^ nuclei (Figure 3C) were reduced compared to WT. The hilus area in WT slices strongly increased between P2 and P6 and declined between P6 and P9 (Figure 3E), thus reflecting the main cellular migration pattern from the neuroepithelium to the DG during early postnatal development, followed by migration from the hilus to GCL (Altman and Bayer, 1990a, 1990b). Although the hilar area at P2 was similar, the increase between P2 and P6 was much less pronounced in *Fbxo41* KO slices, resulting in a smaller hilus at P6 (Figure 3E). In both genotypes, cell number and density in the hilus decreased with time, as previously observed (Figures 3 and S2 and Nicola et al., 2015), with slightly lower cell numbers in *Fbxo41* KO slices throughout development (Figure 3F). The cell density in the hilus at P2 and P6 was higher in *Fbxo41* KO compared with WT (Figure 3G). Together, these data show that the hippocampal layering is not affected in *Fbxo41* KO mice during early postnatal development; however, from P6 onward, the DG is smaller with reduced GCL and hilus size and with fewer cells. Finally, the accumulation of cells in the hilus may indicate reduced migration from the hilus to the GCL during early postnatal development.

### Fewer GFAP^+^ radial glia-like and DCX^+^ cells in Fbxo41 KO granule cell layer

**Figure 4:**
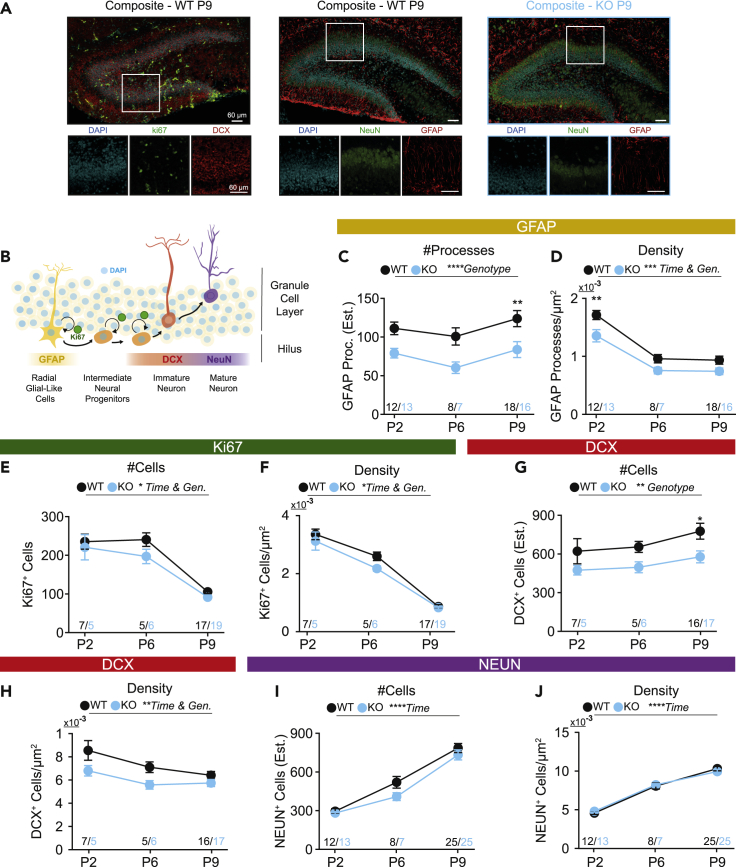
*Fbxo41* KO animals have less GFAP^+^ and DCX^+^ cells in the granule cell layer Immunohistochemistry analysis of WT and *Fbxo41* KO hippocampus at P2, 6, and 9. Estimates of cell/process numbers were obtained by dividing the total intensity of the marker inside a mask by the total intensity of the same marker in a single cell. Density was calculated by dividing cell numbers per area. Note that because of differences in single cell detection, cell numbers between different markers are not comparable. Corresponding analysis in the hilar region can be seen in Figure S4. (A) Example images of WT P9 hippocampal slices stained for DAPI, DCX and ki67 (left), DAPI, NeuN, and GFAP (center) and KO hippocampal slices stained for DAPI, NeuN, and GFAP (right); scale bars 60 μm. (B) Schematic representation of the different markers used. In the DG, mitotic GFAP^+^ radial-glial-like cells (RGLs) generate neuronal precursors. Ki67 is a marker of cell division (Gerdes et al., 1983). Newborn neurons first express markers of immaturity (doublecortin, DCX) and later markers of maturity (NeuN). Scheme adapted from studies on adult neurogenesis (Kempermann et al., 2004, 2015; Nicola et al., 2015). (C) The number of GFAP^+^ processes is significantly smaller in animals lacking Fbxo41. GFAP^+^ cells in the dentate gyrus have their cell body at the hilus while their processes project to the GCL. (D) The density of GFAP processes decreases with time and is significantly smaller at P2 in KO animals compared to WT littermates. (E and F) Both the total number (E) of ki67^+^, as well as density (F) inside the GCL decreases over time, and is slightly reduced in *Fbxo41* KO animals. (G) The number of DCX^+^ cells is smaller in animals lacking Fbxo41. (H) DCX^+^ cells per μm^2^ decreases with time and is lower in the absence of Fbxo41. (I and J) Both the number (I) and density (J) of NeuN^+^ cells increase with time and are not different between WT and Fbxo41 KO. Data is represented as mean ± SEM. Numbers in figures are slices. For statistical details see Table S1.

Next, we tested DG cellular composition using markers of cell division and neuronal maturity (Figures 4A–4B). During neurogenesis, RGL stem cells proliferate into intermediate neural progenitors that mature into neurons (Figure 4B and Goncalves et al., 2016; Kempermann et al., 2004). As previously shown (Nicola et al., 2015), the density of cells expressing a marker of cell division (Ki67) and markers of immaturity (GFAP and DCX) decreased over time in WT slices, whereas mature neuronal markers (NEUN) increased (Figures 4C–4J). Glial fibrillary acidic protein (GFAP) is a widely used marker for RGL cells, which in the mature DG organize in such a way that cell bodies are present in the hilus, whereas radial processes project into the GCL (reviewed in Goncalves et al., 2016; Kempermann et al., 2004). The density of GFAP^+^ processes in the GCL decreased from P2 to P9 in both genotypes (Figures 4C–4D). However, both the number and density of GFAP^+^ processes were lower in *Fbxo41* KO (Figures 4C, 4D, and S3B). As RGL cells proliferate in the GCL, we tested whether the reduction of GFAP^+^ cells correlated with a reduction of Ki67-positive cells (Figures 4E and 4F). *Fbxo41* KO sections had less Ki67^+^ cells and a decreased cell density, although the effect size of this difference was small (Figures 4E and 4F). RGL cells give rise to immature (DCX^+^) and mature (NEUN^+^) neurons (Figure 4B). DCX^+^ cell density decreased over time in both genotypes, as expected (Figure 4H). Both the number and density of DCX^+^ cells were lower in *Fbxo41* KO compared with WT (Figure 4G and 4H). Hence, in *Fbxo41* KO the number of RGL-like cells and immature (DCX^+^) neurons is reduced compared with WT. In the final stage of neuronal maturation, NEUN^+^ neurons integrate into networks (Figure 4B). At P2, NEUN^+^ neurons distributed across the DG and accumulated in the outer edge of the GCL at P6 and P9 (Figures 4A and S3A). The number of NEUN^+^ neurons increased over time similarly in WT and *Fbxo41* KO mice (Figures 4I and 4J) with a slightly smaller NEUN^+^ area at P6 in *Fbxo41* KO (Figure S3C). In conclusion, the number of immature but not mature neurons is decreased in the developing GCL in *Fbxo41* KO.

### Delayed neuronal migration in Fbxo41 KO hippocampus

**Figure 5:**
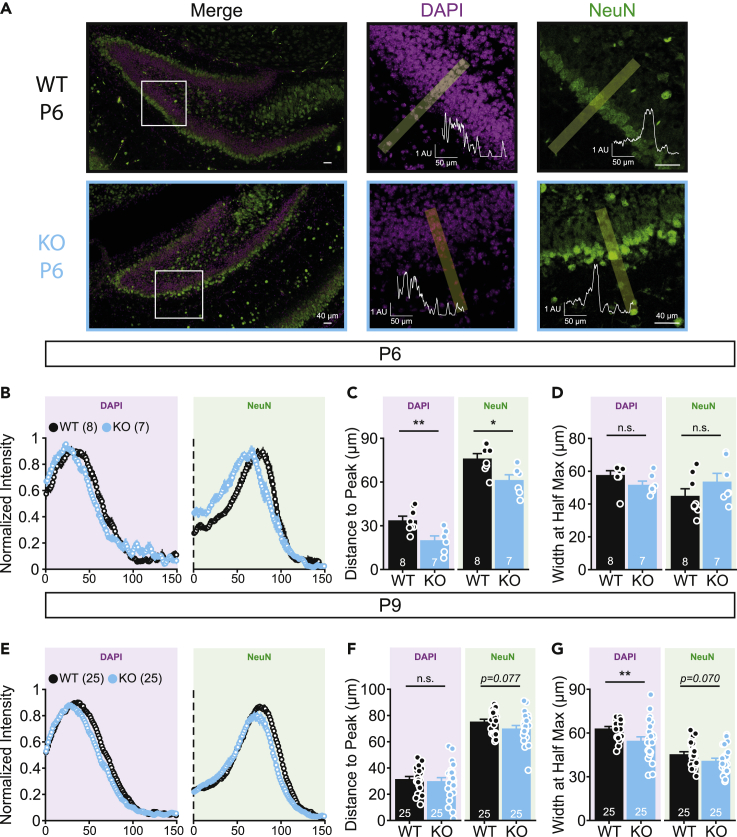
Migration of NeuN^+^ cells is reduced at P6 in DG of *Fbxo41* KO mice The intensity distributions of DAPI and NeuN across the DG were analyzed in P6 and P9 slices. Analysis for the DAPI signal depicted in purple boxes and analysis for the NeuN signal in green boxes. (A) The intensity profiles of NeuN and DAPI were analyzed along lines of 150 μm, starting at the hilus (see STAR Methods section); scale bars 60 μm. Insets depict the intensity profile of DAPI (center) and NeuN (right) across the depicted line in yellow. Example figures show a P6 WT (top) and KO slice (bottom). NeuN positive neurons appear to be somewhat disorganized, although this was not a consistent finding in all slice preparations. (B**–**D) At P6, both DAPI and NeuN intensity profiles are left-shifted (B) with intensity peaks closer to the hilus (C). The width at half maximum is not affected (D). (E**–**G) At P9, distance to peak of the DAPI intensity is similar (F) and width at half maximum (G) is significantly lower in *Fbxo41* KO. Distance to peak of the NeuN intensity (F) and width at half maximum (G) are not statistically different from WT. Data from 3 (P9) or 2 (P6) independent weeks. Data is represented as mean ± SEM. Numbers in figures are slices. For statistical details see Table S1.

To further characterize the effect of FBXO41 in DG development, a similar quantification was performed in the hilus (Figure S4). As observed in the GCL, GFAP^+^ cell number and density were decreased in *Fbxo41* KO (Figures S4A and S4B). In addition, the total number of Ki67^+^, DCX^+^, and NEUN^+^ cells were slightly reduced in *Fbxo41* KO (Figures S4C, S4E, and S4G). Although DCX^+^ and Ki67^+^ cell density was unaffected (Figures S4D and S4F), the density of NEUN^+^ cells at P6 was higher in the hilus in *Fbxo41* KO compared to WT littermates (Figure S4H), similarly to what was observed for DAPI^+^ nuclei (Figure 3G). Mature (NEUN^+^) neurons were present at the hilus at P2 and accumulated at the outer edge of the GCL at P6 and P9 in WT and KO hippocampi (Figures 4A and S3A), in line with canonical neuronal migration patterns in the DG during development (Altman and Bayer, 1990a, 1990b; Urban and Guillemot, 2014). The increase in NEUN^+^ cell density in the hilus at P6 in *Fbxo41* KO (Figure S4H) suggests a delayed neuronal migration from the hilus to the GCL. To test this, intensity profiles of DAPI^+^ and NEUN^+^ cells in the GCL were analyzed (Figure 5A). At P6, the distribution of both NEUN^+^ and DAPI^+^ cells shifted toward the hilar region in *Fbxo41* KO sections, with reduced distance to peak (Figures 5B and 5C). At this stage, the widths of the DAPI^+^ and NEUN^+^ layers were similar in both genotypes (Figure 5D). At P9, the intensity profile and layer width of NEUN^+^ area was similar to WT (Figure 5E), whereas the DAPI^+^ area was not shifted but showed a reduced width in the *Fbxo41* KO sections (Figures 5F and 5G). Hence, these data indicate that cell migration from the hilus to the GCL is delayed in *Fbxo41* KO hippocampi at P6, when cells accumulate closer to the hilus. This observation correlates with the reduced width of the GCL layer at P9.

### Reduced network activity in Fbxo41 KO DG at P6

**Figure 6:**
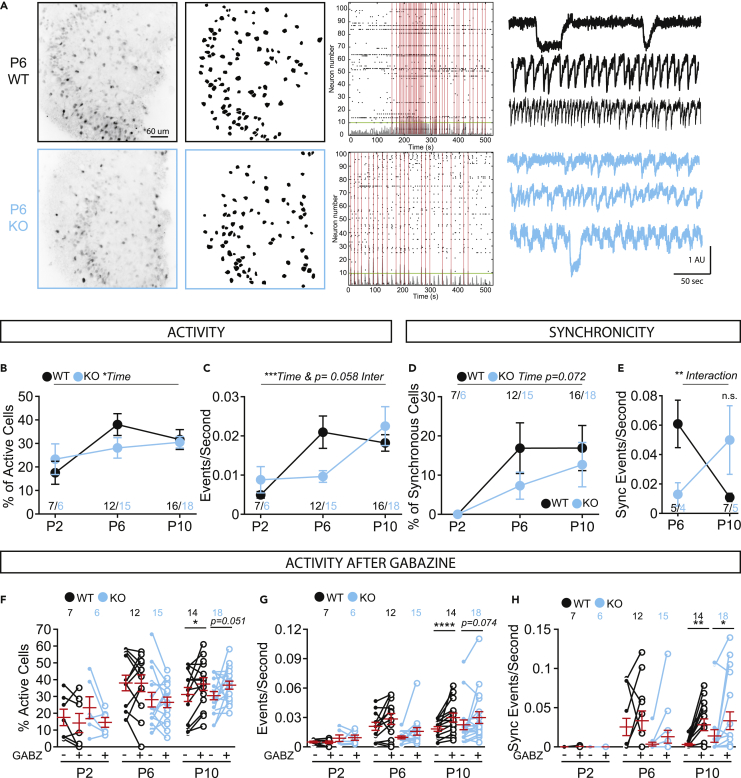
DG network activity is affected at P6 *Fbxo41* KO slices Two-photon calcium imaging in acute DG slices of Fbxo41 KO and WT littermates. (A) Example images of P6 WT (black) and KO (blue) slices. Left panel, collapsed z-stack showing Fura2-labeled neurons (scale bar 60 μm). Middle panel, mask depicting all neurons, and the right panel shows activity measured inside each detected neuron summarized in raster plots showing the cumulative number of active cells with the threshold for synchronous event detection (green line) and synchronous events (red lines). Typical example traces of neuron activity shown on the right. (B) The percentage of active cells increases over time and is not affected in Fbxo41 KO. (C) The frequency of events in active cells increases at P6 in the WT slices, but only at P10 in *Fbxo41* KO. Of note, the difference in number of events per second between WT and KO is only visible at P6, and statistical analysis using a two-way ANOVA has less power to detect effects that occur only at one time point. However, there is an interaction effect between time and genotype (p = 0.058), indicating a delay in increase in events/second and a t-test comparing only differences at P6 is highly significant (p = 0.0097). (D) At P2 no synchronous events are detected in WT and KO slices. Synchronous events increase between P6 and P10 in WT and KO conditions equally. (E) The number of synchronous events per second decreases from P6 and P10 in WT slices and increases in *Fbxo41* KO. (F and G) The percentage of active cells (F) and the number of events per second in active cells (G) increase upon treatment with gabazine at P10 in WT with a trend for increase in the KO condition. (H) At P10, gabazine treatment increases the number of synchronous events both in WT and KO. Gabazine has no significant effect at P2 or at P6. Effect of Gabazine on percentage of synchronous cells upon gabazine treatment is depicted in Figure S5C. Data from 3 (P2/3) and 4 (P6/7 and P10/11) independent experimental weeks. Data is represented as mean ± SEM. On figures F**–**H each individual point represents one slice and the lines connect the measures at baseline (Bas, closed circles) and upon gabazine treatment (10 μm Gbz, open circles) of the same slice. In red is depicted average ± SEM. Numbers in figures are slices. For statistical details see Table S1.

FBXO41 affects neurotransmission in inhibitory and excitatory neurons in culture. We hypothesized that this, together with the delayed neuronal migration, could lead to disturbed network activity that may impact network maturation. Spontaneous network activity during development has been extensively studied using calcium imaging and patterns of activity have been described in several brain areas, including the hippocampus (Crepel et al., 2007). To study these patterns, we labeled hippocampal coronal slices at postnatal day 2-3 (P2), 6-7 (P6), and 10-11 (P10) with the calcium indicator fura2-AM and recorded calcium dynamics in the DG (Figure 6A). Network activity increased in WT slices over time, as observed in the percentage of active cells (Figure 6B) and event frequency (Figure 6C). In *Fbxo41* KO slices, this increase was delayed (Figure 6C). Synchronous events were not detected at P2 in WT or KO slices (Figure 6D). At P6, the number of cells engaging in synchronous firing increased in WT and stabilized at P10 (Figure 6D). In *Fbxo41* KO slices, this increase appeared slower but not significantly different (Figure 6D). However, the frequency of synchronous events increased in KO later than in WT (Figure 6E). Hence, the emergence of synchronous activity, a sign of network maturity, is delayed in *Fbxo41* KO hippocampal slices. Similar to what was reported in the CA1 (Crepel et al., 2007), different types of calcium signals, with different kinetics, were detected in DG (Figure 6A – right panel). FBXO41 deficiency did not affect event duration (Figures S5A and S5B). In WT and KO slices, fast events (<2 s) were absent at P2 and increased to ±5% of events at P10. Most events lasted between 2 and 10 s in both genotypes. In WT, these events increased from P2 to P6 and stabilized at P9; however, in *Fbxo41* KO slices, this percentage was stable at all time points (Figure S5B). In WT and KO slices, the GABA-A receptor antagonist, gabazine, increased the percentage of active cells and the frequency of synchronous events at P10 but not at P2 or P6, as expected (Figure 6F–H and S5C). Taken together, our results show that the occurrence of network synchronicity is delayed in *Fbxo41* KO DG, which correlates with delayed neuronal migration and that GABA affects network activity similarly in both genotypes.

## Discussion

Genetic mutations in E3 ligases have been linked to neuropsychiatric disorders, but a comprehensive understanding of the function of most E3 ligases is lacking. Here, we show that deletion of the SCF-E3 ligase subunit Fbxo41 decreases the efficacy of neurotransmission in cultured neurons, with a more significant impact on GABAergic transmission. In addition, postnatal DG development is disturbed. *Fbxo41* KO mice had a smaller DG (Figure 3) with fewer RGL cells and DCX-positive neurons (Figures 4 and S4). In addition, cell migration to the GCL was disturbed (Figures 3G and 5) and the development of synchronized network activity was delayed (Figure 6). Together, our findings point toward a role for FBXO41 in early postnatal DG development, even before behavioral deficits are detected in *Fbxo41* null mutants.

### FBXO41 affects synaptic transmission in GABAergic and glutamatergic neurons

FBXO41 deficiency resulted in less efficient synaptic communication in striatal inhibitory and hippocampal excitatory neurons, with more significant effects in GABAergic neurons. Based on the extensive body of literature on the protein machinery involved in synaptic vesicle release (reviewed in Sudhof and Rizo, 2011), it is unlikely that FBXO41 is part of the release machinery. Instead, as a substrate recognizing E3 ligase component, FBXO41 may target synaptic proteins for ubiquitination, as previously shown for other E3 ligases (reviewed in Mabb and Ehlers, 2010; Tai and Schuman, 2008). Our data provide several insights into potential FBXO41 targets affecting neurotransmission. First, as neuronal morphology and synapse density are not affected in *Fbxo41* KO neurons, structural or synapse adhesion molecules are unlikely targets. Second, *Fbxo41* KO leads to a reduction in synaptic transmission in glutamatergic and GABAergic neurons. However, the most significantly affected aspects of neurotransmission differ between these two neural populations. The reason may be that there is more variability in the data of glutamatergic hippocampal neurons. Alternatively, FBXO41 might affect different synaptic components of neurotransmission in the two cell types or similar components with slightly different roles in GABAergic and glutamatergic neurons. Third, striatal neurons have lower levels of synaptophysin at synapses and a smaller RRP, indicative of decreased vesicle numbers. Hence, although it remains to be tested if synaptophysin levels are also reduced *in vivo* and whether reduced levels are indeed a proxy for reduced vesicle numbers, FBXO41 may influence vesicle or protein traffic to synapses in striatal neurons. Interestingly, mutations in other E3 ligase components also affect synaptic transmission in excitatory and inhibitory neurons differently (Dong et al., 2020; Helton et al., 2008; Wallace et al., 2012). The mechanisms of such differential regulation are not yet fully understood and substrates not identified, but it has been suggested that these E3 ligases may target proteins that are expressed differently in inhibitory and excitatory neurons (Wallace et al., 2012).

### Fbxo41 KO mice have a smaller DG with reduced GFAP^+^ and DCX^+^ cell populations

In WT mice, the GCL area and cell numbers increased over time, whereas cell density in the hilus decreased (Figure 3), reflecting a previously described model of cell migration from the hilus to the GCL (Altman and Bayer, 1990a, 1990b). In animals lacking FBXO41, the GCL area and cell numbers were similar at P2 but gradually decreased at P6 and P9 compared with WT (Figures 3B and 3C). In contrast, hilar area and cell numbers were already lower at P2, and the typical increase in hilar area at P6 in WT was significantly smaller in KO (Figure 3E). Hence, FBXO41 depletion results in early defects in DG size, well before any observable behavioral phenotype, with reductions in cell numbers in the hilar region preceding those in the GCL.

Characterization of neurogenic markers revealed that the number of RGL cells and immature DCX^+^ neurons were reduced, already at P2 (Figures 4 and S4), whereas mature NEUN^+^ neuron numbers and density in the GCL were unaffected (Figure 4). During DG development, RGL cells migrate and proliferate from the dentate neuroepithelium (Altman and Bayer, 1990a, 1990b; Urban and Guillemot, 2014). Defects in overall cell proliferation, migration, or viability in the neuroepithelium seem unlikely as this would affect overall brain size and organization already at birth, and would also affect the number of NEUN^+^ neurons, which we do not observe. During development, RGL cells divide either symmetrically or asymmetrically to self-renew and originate neurons, respectively (reviewed in Homem et al., 2015). Enhanced asymmetric division of RGL cells in *Fbxo41* KO is in line with the observed phenotype. Enhanced asymmetric division results in increased number of DCX^+^ and NEUN^+^ neuron; however, due to the depletion of proliferative RGL cells, DCX^+^ neuron numbers would reduce over time whereas end-stage mature NEUN^+^ neuron numbers would be less affected, which is in line with our observations. Similar defects of premature depletion of progenitor RGL cells have been previously described to lead to smaller brains (reviewed in Homem et al., 2015), which is also observed for *Fbxo41* KO brains from P12 onwards (Mukherjee et al., 2015). Defects in neural stem cells are a hallmark of microcephaly, a neurodevelopmental disorder in which brains are smaller but with normal brain architecture (reviewed in Thornton and Woods, 2009). Most genes are linked to microcephaly code for centrosomal proteins (reviewed in Thornton and Woods, 2009). Interestingly, primary cilium function is thought to be instructive, at least in part, for asymmetric/symmetric division decisions. FBXO41 shares molecular and phenotypic features with microcephaly genes: it accumulates at centrosomes (King et al., 2019; Mukherjee et al., 2015), and its absence results in a decrease of RGL cell numbers, without affecting the number of mature neurons.

### Cell migration from the hilus to the GCL is delayed in Fbxo41 KO

The hilus transitions from a highly proliferative relatively undefined area during early development to a larger area encaged by the GCL and without cell division in adulthood (Altman and Bayer, 1990a, 1990b; Nicola et al., 2015). During this transition, cells migrate from the hilus to the GCL (Altman and Bayer, 1990a, 1990b). The timing of this transition starts around P5 and is believed to end at P14 in mice (Nicola et al., 2015; Seki et al., 2014). Accordingly, we observed that NEUN^+^ cells accumulate in the hilus at P2; however, at P6, they accumulate at the outer edge of the GCL (Figure S3A). In *Fbxo41* KO at P6, the hilus area was smaller with increased DAPI^+^ nuclei density (Figures 3E and 3G) that coincided with the increased NEUN^+^ cell density at the hilus (Figure S4H) and decreased NEUN^+^ area (Figure S3C). We hypothesized that these resulted from defective migration of cells from the hilus to the GCL. Indeed, at P6, the cells accumulated closer to the hilus (Figures 5B and 5C), and at P9, the GCL width was reduced (Figure 5G). These observations are in line with a delay in radial migration in the GCL between P6 and P9 and consistent with a previous study showing that FBXO41 depletion results in defective cell migration and delayed layer organization in the cerebellum at P16 (Mukherjee et al., 2015).

### Developmental network activity is affected in the absence of Fbxo41

A final stage in DG development is the establishment of active neuronal networks. In *Fbxo41* KO DG, the increase in network frequency was delayed (Figure 6C), which also affected the appearance of synchronous events (Figure 6E). Taken together, our data suggest that the absence of FBXO41 delays the fine-tuning of network activity, which is relevant given that correctly timed development of network activity is important for mature network activity and development of sensory maps (Che et al., 2018; Kirkby et al., 2013).

In summary, our study shows that FBXO41 controls DG growth and composition, migration from hilus to GCL, and network activity maturation. Our experiments indicate that FBXO41 may regulate RGL cell division, which fits with its centrosomal location. The migration deficits and network activity delays in *Fbxo41* KO could be explained by defects in centrosomal or ciliary function and hence suggest a uniform role of FBXO41 affecting multiple pathways, possibly through targeting a small set of centrosomal proteins for ubiquitination. Hence, FBXO41 emerges as a candidate to modulate brain development and synaptic activity.

### Limitations of the study

Although our study uncovers new insights into the function of Fbxo41, we do not identify targets of the E3 ligase. Studies aimed to uncover these targets are underway and will be instrumental to further our understanding of Fbxo41’s role in synaptic transmission and brain development. Limitations of the current study are the fact that our single cell measurements were slightly underpowered to detect small effect sizes in synaptic transmission.

## STAR★Methods

### Key resources table

**Table:** 

REAGENT or RESOURCE	SOURCE	IDENTIFIER
**Antibodies**		

Actin	Chemicon	Cat# MAB1501; RRID: AB_2223041
Fbxo41	ProteinTech	Cat# 24519-1-AP; RRID: AB_2879586
MAP2	Abcam	Cat# ab5392; RRID: AB_2138153
SMI-312	Biolegend	Cat# Smi-312R; RRID: AB_2314906
Synaptophysin 1	Synaptic Systems	Cat# 101 011; RRID:AB_887824
GFAP	Synaptic Systems	Cat# 173004; RRID: AB_10641162
NeuN	Millipore	Cat# ABN78; RRID: AB_10807945

**Chemicals, peptides, and recombinant proteins**		

SIGMA*FAST*™ Protease Inhibitor	Sigma-Aldrich	Cat# S8820
poly-D-lysine	Sigma	Cat# P6407
Glutamax	GIBCO	Cat# 31966-021
L-cysteine	Sigma	Cat# C7352
CaCl_2_	Sigma	Cat# C7902
EDTA	AppliChem	Cat# A2937.0500
Fetal Bovine Serum	Gibco	Cat# 10270
Albumine	Applichem	Cat# A1391.0100
Trypsin Inhibitor	Sigma	Cat# T9253
Non-essential aminoacids	Sigma	Cat# M7145
Penicillin-Streptomycin	Gibco	Cat# 15140-122
Hanks’ balanced salt solution	Sigma	Cat# H9394
HEPES	Gibco	Cat# 15630-056
Trypsin	Gibco	Cat# 15090-046
Neurobasal medium	Gibco	Cat# 21103-049
B-27	Gibco	Cat# 17504-044
Glutamax	Gibco	Cat# 35050-038
Formaldehyde	Electron Microscopies Sciences	Cat# 15680
Triton X-100	Fisher Chemical	Cat# T/3751/08
Normal Goat Serum	Gibco	Cat# 16210-072
Mowiol 4-88	Sigma	Cat# 81381

**Experimental models: Organisms/strains**		

Rat: Wistar (Crl:WI)	Charles River	Strain code: 003

**Software and algorithms**		

SynD – Synapse and neurite detection	Schmitz et al., 2011	https://www.johanneshjorth.se/SynD/SynD.html
ImageJ	Ferreira et al., 2015	https://imagej.net/Welcome RRID:SCR_003070
MATLAB R2018a	MathWorks	https://www.mathworks.com
GraphPad Prism	GraphPad Software	https://www.graphpad.com/ RRID:SCR_002798

### Resource availability

#### Lead contact

Further information and requests for resources and reagents should be directed to and will be fulfilled by the Lead Contact, Ruud Toonen (ruud.toonen@cncr.vu.nl).

#### Materials availability

This study did not generate new unique reagents.

### Experimental model and subject details

*Fbxo41* heterozygous mice described previously (Mukherjee et al., 2015) were used for timed mating to obtain homozygous *Fbxo41* wildtype and knockout mice. All newborn mice used for experiments were genotyped by PCR. For glia preparations newborn pups from female Wistar rats were used. Animals were housed and bred according to institutional and Dutch governmental guidelines (DEC-FGA 11-03 and AVD112002017824).

### Method details

#### Western Blot

To test FBXO41 expression levels at different postnatal days and brain areas, brain samples were isolated at different time points, weighted and triturated in ice-cold PBS (phosphate-buffered saline, 137 mM NaCl, 2.7 mM KCl, 6.5 mM Na_2_HPO_4_, 1.5 mM KH_2_PO_4_) with protease inhibitors (SIGMAFAST™ Protease Inhibitor; Sigma-Aldrich). Samples were centrifuged for 5 min at 4°C, the supernatant discarded and 100 μL of Laemmli sample buffer added per 0.01 g of brain (2% w/v sodium dodecyl sulfate (SDS), 10% v/v Glycerol, 0.26 M β-mercaptoethanol, 60 mM Tris-HCl pH 6.8, and 0.01% w/v Bromophenolblue). Samples were heated for 5 min at 90°C, then homogenized using insulin needles, and kept at -20°C until further use. Samples were boiled for 5 min at 90°C, homogenized and loaded on an 8% SDS-polyacrylamide gels with 2,2,2-Trichloroethanol and transferred to Polyvinylideenfluoride (PVDF) membranes (Bio-rad) (1 h, 0.3 mA, 4°C). Blots were blocked in a solution of PBS containing 0.1% Tween-20, 2% milk and 0.5% normal goat serum, and incubated with primary antibodies overnight at 4°C under medium agitation. The following antibodies were used, all dissolved in blocking solution: monoclonal mouse anti Actin (1:10,000; Chemicon, MAB1501), polyclonal rabbit anti FBXO41 (1:500; ProteinTech, 24519-1-AP). Blots were washed with PBS containing 0.1% Tween-20 and then incubated with alkaline phosphatase conjugated secondary antibodies (dissolved in blocking solution) for 1 h at room-temperature, in the dark and under medium agitation. After blots were washed in PBS containing 0.1% Tween-20, incubated for 5 min with AttoPhos (Promega) and scanned using a FLA-5000 fluorescent image analyzer (Fujifilm).

#### Neuronal micro-dot culture

Glial micro-islands were obtained as followingly described (Toonen et al., 2006; Wierda et al., 2007). Briefly, glass coverslips were etched in 1M HCl for at least 2 h, followed by washes with water, and 1 h incubation in 1M NaOH. Coverslips were sterilized in 70% EtOH, put in 12-well plates and treated with 1.5 mg/mL Agarose for 30 min. Afterwards, coverslips were stamped with poly-D-lysine (Sigma, P6407)-collagen (0.5 mg/mL, 3.66 mg/mL collagen in 17 mM acetic acid), and sterilized with UV light for 20 min. Glia cells were obtained from P0-1 rat pups. Rat cerebral cortex was incubated 45 min at 37°C with Enzyme Solution (DMEM + Glutamax (Gibco, 31966-021)), 0.2 mg/mL of L-cysteine (Sigma, C7352), 100 mM CaCl_2_ (Sigma, C7902), 50 mM EDTA (AppliChem, A2937.0500) and 20-25 units/mL of papain; solution was bubbled with carbogen and filtered before use), and then incubated for 15 min at 37°C with inhibitor solution (DMEM + Glutamax, 10% heat-inactivated fetal bovine serum (Gibco, 10270), 2.5 mg/mL Albumine (Bovine Fraction V; Applichem, A1391.0100) and 2.5 mg/mL Trypsin Inhibitor (Type II-O, Chicken Egg; Sigma, T)). Solution was replaced with DMEM culture medium (DMEM + Glutamax, 10% heat-inactivated fetal bovine serum, 1x non-essential aminoacids (Sigma M7145), and 1x and 0.1% Penicillin-Streptomycin (Gibco, 15140-122)). Cells were triturated with a fired polished pasteur pipette, counted and plated at a density of 8000/well.

Neurons were obtained from *Fbxo41* KO P1 pups, after genotype was confirmed, from either hippocampus or striatum. Isolation of both neuronal types was similar. Brains were isolated in Hanks’ balanced salt solution (Sigma, H9394) supplemented with 10 mM HEPES (Gibco, 15630-056) and hippocampi and striatum removed. They were then incubated 0.25% Trypsin (Gibco, 15090-046) in Hanks-HEPES for 20 min at 37°C. Trypsin was removed with 3x washes of Hanks-HEPES solution and triturated in DMEM culture medium with a fired polish pasteur pipette. Triturated cells were spun down for 5 min at 800 rpm, the pellet resuspended in Neurobasal medium (Gibco, 21,103-049) supplemented with 2% B-27 (Gibco, 17,504-044), 1.8% HEPES, 0.25% glutamax (Gibco, 35,050-038) and 0.1% Penicillin-Streptomycin, and cells counted. Hippocampal and striatal neurons were plated in 12-well plates at density of 1400 cells/well on 18 mm coverslips with rat glia micro-dot cultures prepared 3 days before.

#### Immunocytochemistry and confocal microscopy

Neurons were fixed at DIV 14 using 3.7% formaldehyde (Electron Microscopies Sciences, 15,680) for 15 min at room temperature. Neurons were washed with PBS, and permeabilized for 8 min at room temperature using PBS containing 0.2% Triton X-100 (Fisher Chemical, T/3751/08). Afterwards neurons were incubated for 40 min in blocking solution (PBS containing 2% normal goat serum (Gibco, 16210-072) and 0.1% Triton X-100) and then for 2 h in primary antibodies, both at room temperature. The following primary antibodies were used, all diluted in blocking solution: polyclonal chicken anti MAP2 (1:20000, Abcam, ab5392), monoclonal mouse anti SMI-312 (1:1000, Biolegend, Smi-312R), polyclonal guinea pig anti synaptophysin 1 (1:1000, Synaptic Systems, 1011004). Coverslips were washed in PBS and incubated in secondary antibody. Secondary antibodies were conjugated with AlexaFluor dyes (1:1000, Invitrogen) and diluted in blocking solution. Finally, coverslips were washed, mounted in Mowiol-Dabco (Invitrogen) and kept at 4°C. Image acquisition was performed with a confocal A1R microscope (Nikon) using a 40x oil immersion objective (NA = 1.3). Images were acquired as single z stack and neuronal morphology was analyzed blindly using automated image analysis software (Schmitz et al., 2011)

#### Electrophysiological recordings

Electrophysiological recordings were obtained at room temperature from DIV13-18 isolated neurons, using whole-cell voltage clamp (holding potential −70 mV) (Wierda et al., 2007). Neurons were kept in a bath of extracellular solution containing 140 mM NaCl, 2,4 mM KCl, 4 mMCaCl_2_, 4mM MgCl_2_, 10 mM HEPES and 10 mM Glucose (pH = 7.3 and Osmolarity 300). The intracellular solution used in the patching pipettes contained 125 mM K^+^-Gluconic acid, 10 mM NaCl, 4 ,6mM MgCl, 4 mM K2-ATP, 15 mM Creatine Phosphate, 20U/mL Phosphocreatine Kinase and 1 mM EGTA. (pH = 7.3 and Osmolarity 300). Stimulus were induced by a 30 mV depolarization of 0.5 ms, and given in increasing order of magnitude. In glutamatergic neurons, spontaneous recordings were followed by paired pulse measurements, 10 Hz train and finally a 40 Hz train with recovery pulses. In GABAergic neurons, spontaneous recordings were followed by single EPSC, paired pulse measurements, and finally a 20 Hz train with recovery pulses. The experimenter was blinded to genotype during recordings and recordings were only analyzed when the estimated series resistance was lower than 12 mΩ and leak current lower than 500pA, and cells responded to all protocols. Spontaneous events were only considered for amplitude analysis when more than 5 events could be detected per cell. First EPSC was calculated form the single EPSC in the striatal neurons, and the first pulse of the 10 Hz train in the hippocampal neurons. The readily releasable pool was estimated by back-extrapolation of the cumulative charge from the last 30 pulses of 100 pulses at 40 Hz or 20 Hz train. Release probability was calculated by dividing the first charge of the train used to calculate the RRP, by the total RRP charge. Recordings were obtained using a MultiClamp 700B amplifier, Digidata 1440 A, and pCLAMP 10.3 software (Molecular Devices). Data was analysed using a custom-written Matlab program R2017a (Mathworks).

#### Immunohistochemistry

Animals were genotyped and pairs of WT and KO littermates were selected. Brains were removed at the indicated postnatal days (P2, P6-7 and P9), and left in 4% formaldehyde solution for at least one week at 4°C. Brains were then washed in PBS (phosphate-buffered saline, 137 mM NaCl, 2.7 mM KCl, 6.5 mM Na_2_HPO_4_, 1.5 mM KH_2_PO_4_, pH = 7,4) and immersed in a 30% sucrose solution (Serva, dissolved in PBS) at 4°C. After brains sunk, they were washed in PBS and kept at −80°C, until slicing. Brains were sliced in 30-35 μm coronal sections using a cryostat and kept in PBS containing 0.02% NaN_3_ until stained. For immunostaining, a fixed number of slices per group was used, to avoid different antibody availability, and procedures were done under slight agitation. Slices were washed with PBS, and then incubated for 30 min with 3% H_2_O_2_ (Merck, dissolved in PBS). Slices were then washed with PBS and blocked for 1 h at room temperature with blocking solution. For immunostainings containing primary goat antibodies the blocking solution was 0.25% fish gelatin (Aurion) and 0.2% Triton X-100 (Fisher Scientific) in PBS, and for all others the blocking solution was 5% normal goat serum (NGS, Fisher Scientific), 2.5% bovine serum albumin (BSA, Fisher Scientific) and 0.2% Triton X-100 (Fisher Scientific) in PBS. Slices were then left overnight at 4°C with primary antibody, washed in PBS and incubated with secondary antibody for 2 h at room temperature in the dark. Slices were then incubated with 4′,6-diamidino-2-phenylindole (DAPI, Invitrogen) diluted in PBS (6.67 μg/mL) for 10 min at room temperature. Slices were mounted on microscope slides with Mowiol-Dabco (Invitrogen) and kept at 4°C. The following primary antibodies were used: guinea pig anti-glial fibrillary acidic protein (GFAP, 1:1000, Synaptic Systems, Cat. N. 173004), rabbit anti-NEUN (1:500, Millipore, Cat. N. ABN78), goat anti-Doublecortin (DCX, 1:1000, Santa Cruz), rabbit anti-ki67 (1:500, Santa Cruz). The following secondary antibodies were used at 1:500 dilution: Alexa Fluor 488-conjugated goat anti rabbit, Alexa Fluor 546-conjugated goat anti guinea pig, Alexa Fluor 488-conjugated donkey anti rabbit, Alexa Fluor 546-conjugated donkey anti goat. Image acquisition was performed with a confocal A1R microscope (Nikon) using a 40x oil immersion objective (NA = 1.3). Images were acquired as z stacks (20 steps of 0.5 μm), and adjacent images were stitched using the NIS software, in order to obtain the image of the full dentate gyrus.

#### Image analysis

The experimenter was blind during image analysis, and the order of images analyzed was randomized. For image-analysis z-stacks were selected for analysis using Fiji Software (Schindelin et al., 2012): for the NEUN, DCX and DAPI signal a single image was used, for GFAP a maximum projection of 4 slices was used (as GFAP processes are not straight). The dentate gyrus and hilus masks were made using the DAPI signal. At P6 and P9, NEUN^+^ cells accumulated at the edge of GCL layer, and were not included inside the GCL area defined using DAPI signal. So, for P6 and P9 a NEUN mask was created using the NEUN signal and in a similar way that the GCL mask was created. To enhance detection of the borders, images were blurred with a gaussian blur. After, area, mean and total intensities of DAPI, NEUN, GFAP, DCX were measured inside the selected masks. NEUN intensities were measured inside GCL mask at P2, and inside NEUN mask at P6 and P9, as at P2, NEUN layer is not yet organized in the outer edge of the GCL. To normalize the signal in each slice, the intensity of single NEUN^+^, GFAP^+^ and DCX^+^ cells were calculated, by averaging the intensity of 5 regions of interest centered on a single neuron per cell-type. The region of interests used were constant in size. The total number of cells was estimated by dividing the total fluorescence of a marker, by the total intensity inside a single-cell (region of interest). Due to small differences in single cell detection accuracy, cell numbers between different markers are not comparable. The cell density was calculated by dividing the cell number by the area. Ki67^+^ cells were mostly non-overlapping and were counted using all z-stacks available. The number of GFAP processes per slice was determined by drawing 2-4 150 μm wide lines per slice parallel to the DG. A line profile was obtained for each line, and the number of peaks assessed using Find Peaks, in BAR plugin in ImageJ (Ferreira et al., 2015). For the distribution of DAPI and NEUN profiles, 10 lines of 150 μm per slice were drawn perpendicular to dentate gyrus, starting at the hilus. For each of these the intensity profile along the line was obtained and an average intensity profile per slice calculated. Normalization of the data was obtained by averaging the maximum 3 and minimum 3 intensity values in each line. Then, the minimum was subtracted and the result divided by the maximum. Finally, both the peak distance from hilus and the width at half maximum were calculated using the average intensity profile per slice. For the peak distance from hilus, the 3 maximum intensity values were detected and the correspondent x values (distance from hilus) averaged. The width at half maximum was calculated as the distance between the curve points at the peak half maximum level. In some of the DAPI intensity profile curves all the points before the peak were higher than width at half maximum. In such cases the first point was defined as x = 0.

#### Calcium imaging

Mice were genotyped, selected and quickly decapitated, and processed s (Hjorth et al., 2016; Dawitz et al., 2011). Animals were collected in three age groups: P2 and P3 (P2/3), P6 and P7 (P6/7), P10 and P11 (P10/11). In each of these groups, one randomly assigned animal was processed each day. Brains were isolated and sliced on ice bathed in iced-ACSF solution containing choline (110 mM Choline chloride, 26 mM NaHCO_3_, 10 mM Dglucose, 11.6 mM sodium ascorbate, 7 mM MgCl_2_, 3.1 mM sodium pyruvate, 2.5 mM KCl, 1.25 mM NaH_2_PO_4_, and 0.5 mM CaCl_2_). Slices of 300 μm were obtained using a vibratome, and were left for at least 30 min at room temperature in resting solution (ACSF solution with higher magnesium concentration: 125 mM NaCl, 26 mM NaHCO_3_, 10 mM D-glucose, 3 mM KCl, 2.5 mM MgCl_2_, 1.6 mM CaCl_2_, and 1.25 mM NaH_2_PO_4_). From this step on slices were bubbled with 95% Oxygen and 5% CO_2_. Slices were transferred to an interface chamber and temperature was slowly raised to 37°C, while in elevated magnesium ACSF. When at 37°C, the slices were loaded with calcium dye (50 μg of fura2-AM, Invitrogen) diluted in 9 μL DMSO and 1 μL Pluronic F-127 (20% solution in DMSO, Invitrogen), between 20 and 40 min depending on the age of the animal. After, the excess dye was washed off for 1 h before transfer to a recording chamber. During image acquisition slices were perfused with oxygenated standard ACSF. Images were acquired using a two-photon microscope (Trimscope, LaVision Biotec) using a Ti-sapphire laser tuned to 820-nm wavelength): first a Z-stack of the selected area was acquired, for detecting cells, and then a 7.65 Hz movie of 8 min was recorded (Dawitz et al., 2011). Analysis was done using previously described MATLAB scripts (Hjorth et al., 2016).

### Quantification and statistical analysis

Statistical analysis was performed using GraphPad Prism 8.0 software, and all statistical details can be found in Table S1. Normality was tested using D’Agostino & Pearson test. A p value of ≤0.05 was considered statistically significant. The following code was used to signify significance: ∗ ≤0.05; ∗∗ ≤0.0021; ∗∗∗ ≤0.0002; ∗∗∗ ≤0.0001. Detailed information per dataset (average, SEM, n and detailed statistics) is shown in Table S1.

## Data Availability

The data that support the findings of this study, as well as any additional information required to reanalyze the data reported in this paper, are available from the Lead Contact upon reasonable request. This paper does not report original code.

## Supplemental information

Document S1. Figures S1–S5

Table S1. Statistical details from data presented, related to Figures 1, 2, 3, 4, 5, 6, S1, S3, S4, and S5
