# Supplementary material for: Neuronal F-Box protein FBXO41 regulates synaptic transmission and hippocampal network maturation
Source: iScience. 2022 Mar 18;25(4):104069. doi: 10.1016/j.isci.2022.104069 (PMC8971942; doi:10.1016/j.isci.2022.104069)
Supplement: Document S1. Figures S1–S5 [file mmc1.pdf]

iScience, Volume *25*

Supplemental information

Neuronal F-Box protein FBXO41 regulates synaptic transmission and hippocampal network maturation

Ana R.A.A. Quadros, Rocío Díez Arazola, Andrea Romaguera Álvarez, Johny Pires, Rhiannon M. Meredith, Ingrid Saarloos, Matthijs Verhage, and Ruud F. Toonen

Supplementary Figures

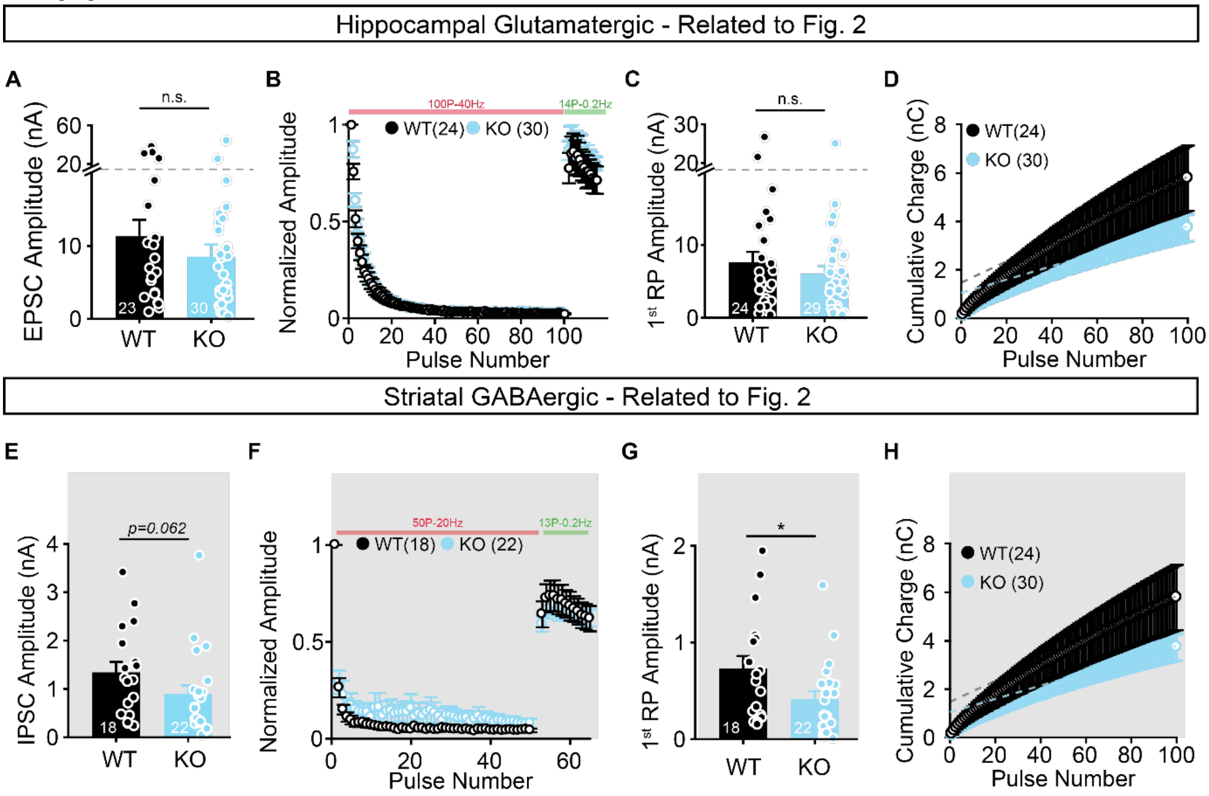

Supplementary Figure S1 – Charge transfer during 20Hz stimulation is lower in *Fbxo41* KO neurons compared to WT littermates, related to Figure 2.

Voltage clamp recordings from the same neurons plotted in Figure 2. Hippocampal Glutamatergic neurons (top) and Striatal GABAergic neurons (bottom, gray boxes).

(A) EPSC amplitude of the first pulse of a 10Hz train is not affected by Fbxo41 deficiency (same neurons as depicted in Fig. 2D)

(B) Normalized amplitude during repetitive stimulation (depicted in Fig. 2E) shows that release kinetics are similar between WT and KO neurons, and that amplitude recovers to WT levels after depletion of the RRP (recovery pulses).

(C) The amplitude of the first recovery pulse after RRP depletion is not affected in *Fbxo41* KO neurons.

(D) Cumulative charge release during the 100 action potentials at 40Hz (depicted in Fig. 2E). Dashed lines represent linear fits to the last 25 action potentials to back extrapolate to y-intercept to calculate the RRP.

(E) IPSC amplitude of a single action potential is not significantly different between neurons lacking FBXO41 and WT control (same neurons as depicted in Fig. 2D).

(F) Normalized peak amplitude of same traces (depicted in Fig. 2E) shows that kinetics of charge transfer is similar between KO and WT neurons throughout the train.

(G) The amplitude of the first recovery pulse after RRP depletion is smaller in KO neurons; however, when the amplitude is normalized to the first pulse there is no differences in recovery between WT and KO (depicted in F).

(H) Cumulative charge release during the 50 action potentials at 20Hz (depicted in Fig. 2E). The lines represent the back extrapolation to calculate the RRP.

Related to Fig. 3

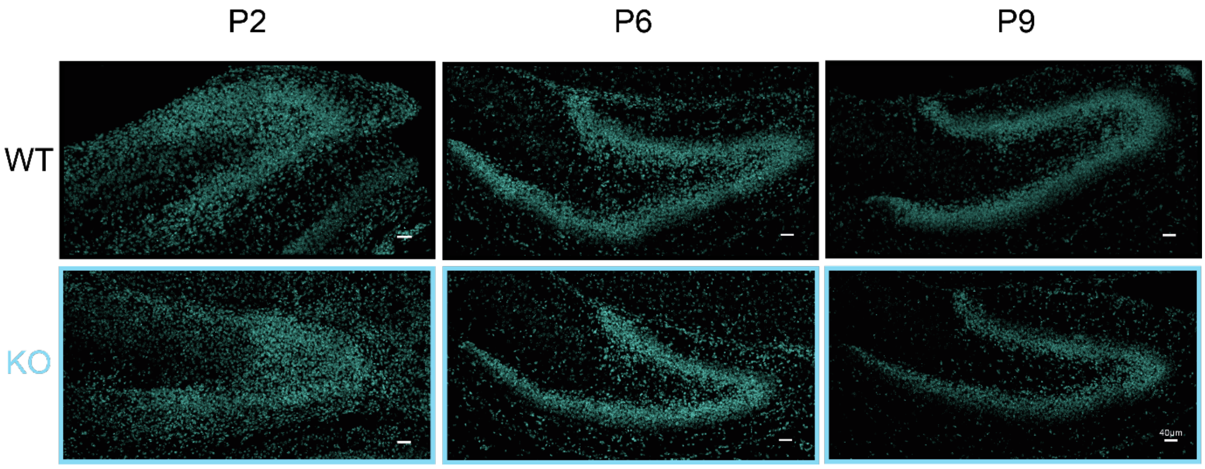

Supplementary Figure S2 – The DG is more organized at P9, compared to P2, related to Figure 3. Example figures of DAPI staining in the DG of P2, P6 and P9 WT (top) and KO animals (bottom); scale bar 40μm. Slices used for analysis of data presented in Fig. 3.

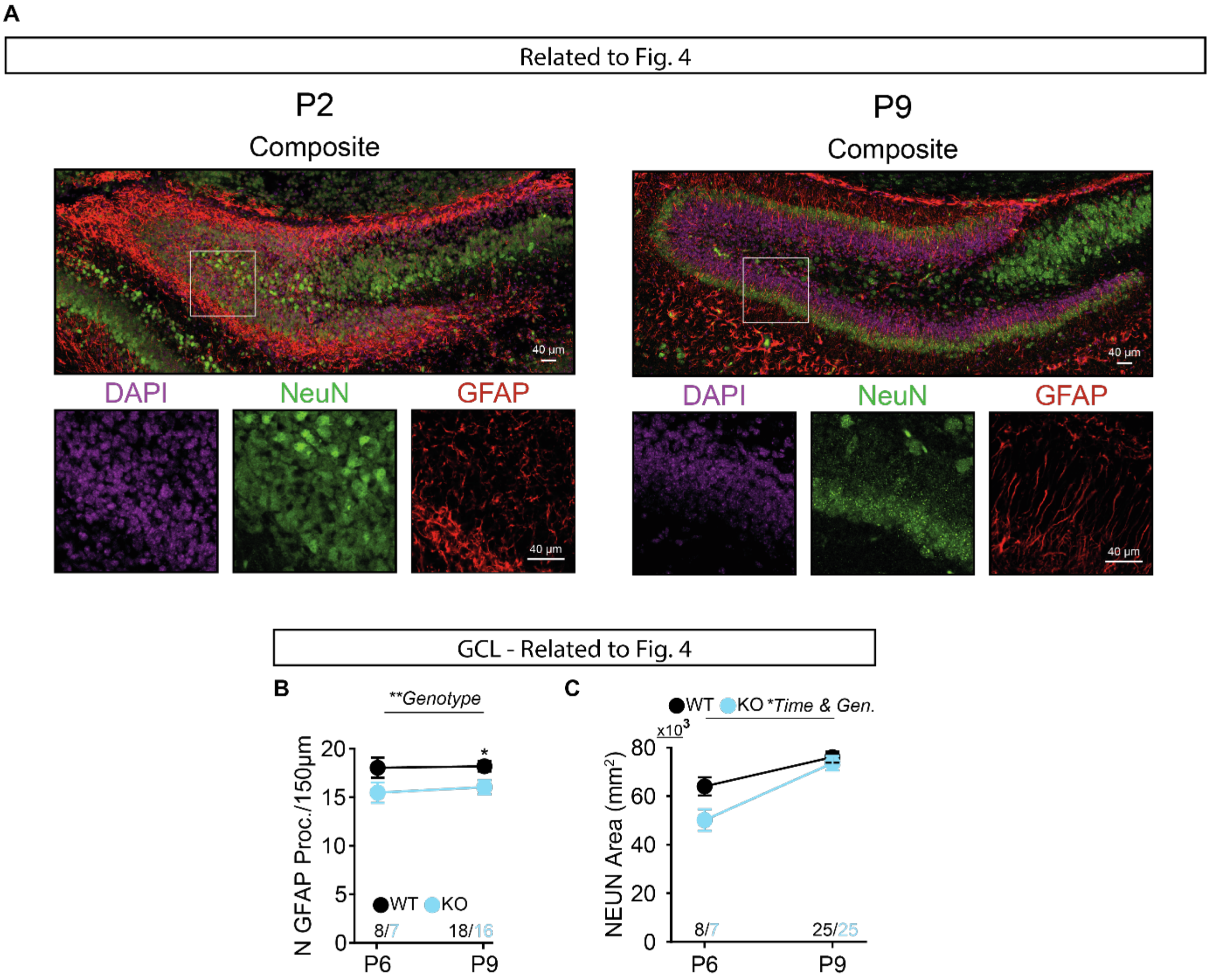

Supplementary Figure S3 – NeuN area is smaller in Fbxo41 KO at P9, related to Figure 4.

(A) Examples of immunostained WT slices at both postnatal days 2 and 9. The top left composite image of a P2 WT slice is stained for DAPI, NeuN and GFAP, and bottom left is a zoom of the individual markers. A P9 WT slice is depicted on the right. At P9 the cell layers in the DG are more compact (DAPI staining). In addition, at P9 GFAP processes organize consistently parallel to the DG, extending from the hilus to the molecular layer, whereas NeuN+ neurons accumulate at the outer edge of the DG. Neither of these organizational features is present at P2, which was also observed previously(Nicola et al., 2015). Slices used for analysis presented in in Fig. 4.

(B) The number of GFAP processes in 150 µm line profiles are smaller at P6 and P9 in animals lacking Fbxo41.

(C) The area, occupied by $NeuN^{+}$ cells, increases from P6 to P10 and is smaller in KO at P6.

Same slices analyzed in Fig. 4. Data is represented as mean ± SEM. Numbers in figures are slices. For statistical details see Supplementary Table 1.

HILUS - Related to Fig. 4

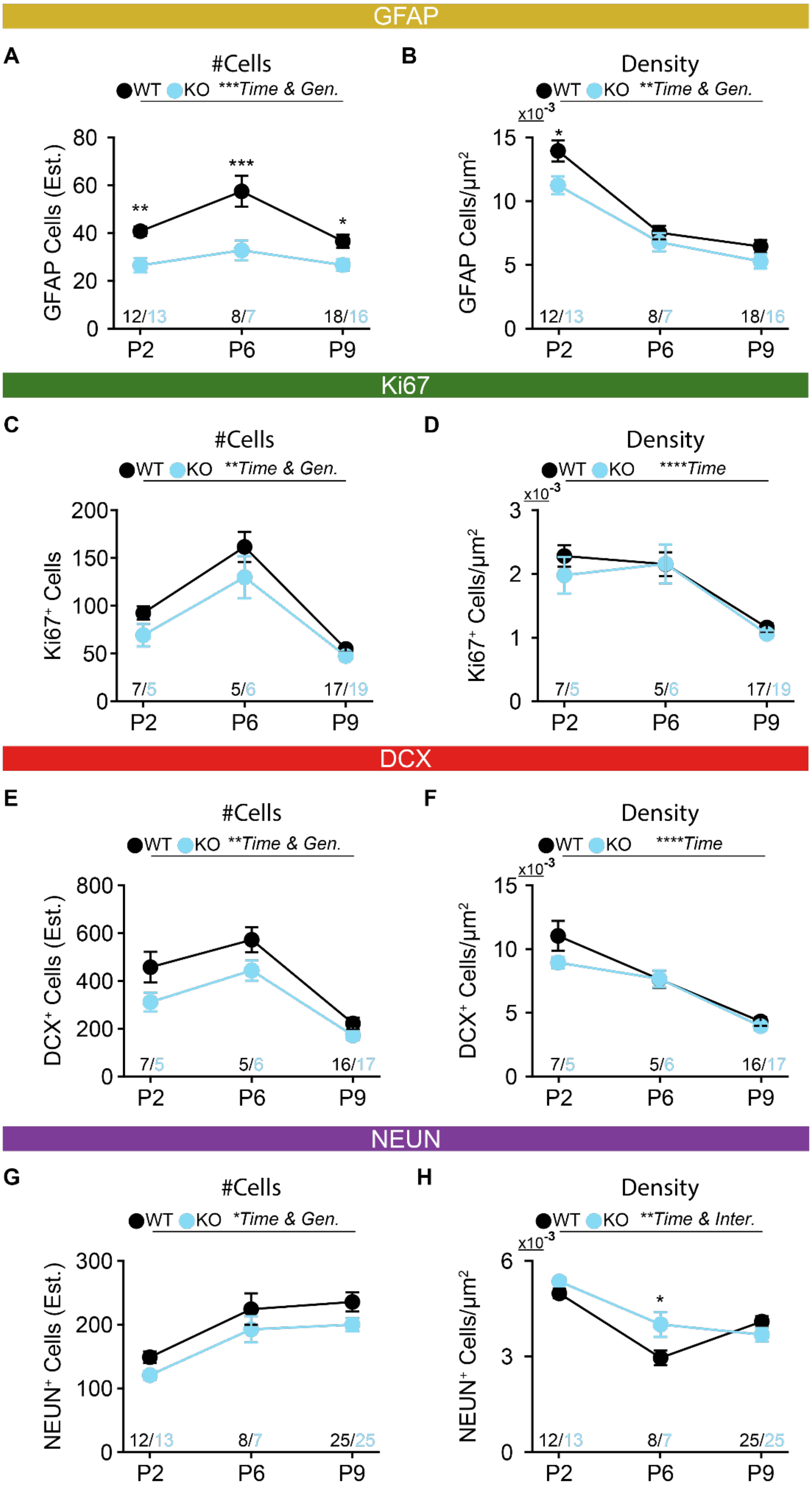

Supplementary Figure S4 – Less GFAP$^+$ and DCX$^+$ cells in *Fbxo41* KO hilar region with increased NeuN$^+$ cell density at P6, related to figure 4.

Immunohistochemistry analysis of hilar region of WT and *Fbxo41* KO hippocampus at P 2, 6, and 9. Slices used for analysis are the same used in Fig. 4.

(**A-B**) Both the total number of GFAP$^+$ cells (**A**), and the GFAP density (**B**) in the hilus are decreased in the absence of FBXO41, already at P2, and decrease with time in both genotypes. GFAP$^+$ cells in the dentate gyrus have their cell body at the hilus while their processes project to the GCL.

(**C**) The total number of ki67$^+$ cells inside the hilus changes over time and is reduced in *Fbxo41* KO.

(**D**) The density of ki67$^+$ cells inside the hilus changes over time, and is not affected in *Fbxo41* KO.

(**E**) The number of DCX$^+$ cells in the hilus, decreases from P2 to P9 and is smaller in *Fbxo41* KO.

(**F**) DCX density decreases with postnatal age in the hilus and is not affected in Fbxo41 KO.

(**G**) The total number of NeuN$^+$ cells in the hilus increases over time and is smaller in *Fbxo41* KO.

(**H**) NeuN density is affected by time and changes differently over time in WT and KO hilar region. At P6, NeuN density is significantly higher in animals lacking FBXO41.

Data is represented as mean ± SEM. Numbers in figures are slices. For statistical details see Supplementary Table 1.

CAICIUM IMAGING - Related to Fig. 6

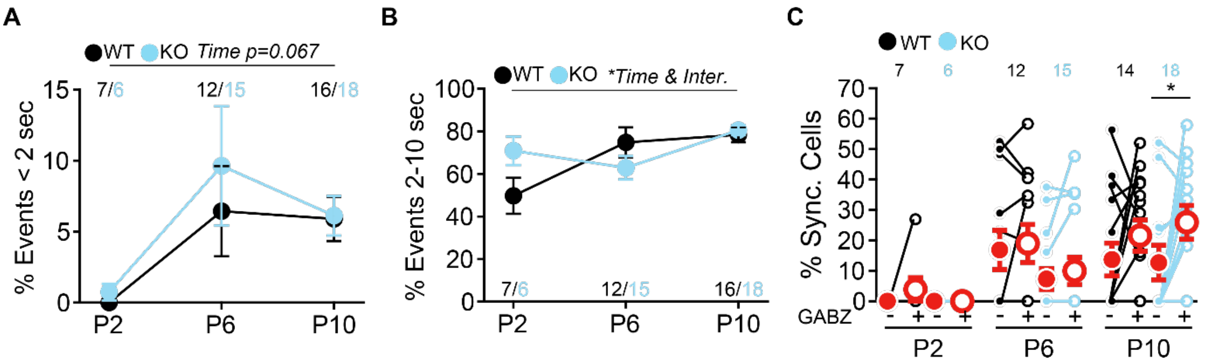

Supplementary Figure S5 – Event duration is mostly unaffected in the absence of FBXO41, related to Figure 6.

Two-photon calcium imaging in acute DG slices of *Fbxo41* KO and WT littermates. These data correspond to events depicted in Fig. 6.

(A-B) Events become faster with age: events tend to last less than 2 seconds at P6-P10 compared to P2 (**A**), and at P10 there are almost no events longer than 10 seconds **(B)**. Change in event duration over time occurs differently in WT and KO.

(C) At P10, gabazine treatment increases the percentage of synchronized cells only in the KO condition. Gabazine has no significant effect at P2 or at P6.

Data from 2-4 independent weeks. Data is represented as mean ± SEM. On figure C each individual point represents one slice and the lines connect the measures at baseline (Bas, closed circles) and upon gabazine treatment (10µm Gbz, open circles) of the same slice. In red is depicted average ± SEM. Numbers in figures are slices. For statistical details see Supplementary Table 1.
